# Danggui Shaoyao San and disassembled prescription: neuroprotective effects via AMPK/mTOR-mediated autophagy in mice

**DOI:** 10.1186/s12906-024-04588-x

**Published:** 2024-08-10

**Authors:** Xiaoqing Cheng, Yuqiong Dai, Baoling Shang, Shuting Zhang, Liting Lin, Qingguang Wu, Ruoting Zhan, Shengqing Li, Sijun Liu

**Affiliations:** 1 School of Pharmaceutical Sciences, Guangzhou University of Chinese Medicine, Waihuan Road, Guangzhou Higher Education Mega Center, No. 232, Guangzhou, 510006 Guangdong China; 2 Second Clinical Medical College, Guangzhou University of Chinese Medicine, Guangzhou, China; 3 The Second Affiliated Hospital of Guangzhou University of Chinese Medicine, Guangdong Provincial Hospital of Chinese Medicine, Guangzhou, China; 4 Science and Technology Innovation Center, Guangzhou University of Chinese Medicine, Guangzhou, China; 5 Key Laboratory of Chinese Medicinal Resource from Lingnan, Guangzhou University of Chinese Medicine, Guangzhou, China

**Keywords:** Alzheimer’s disease, Danggui Shaoyao San, Autophagy, AMPK/mTOR

## Abstract

**Background:**

Danggui Shaoyao San (DSS), a frequently prescribed Chinese medicine formula, has demonstrated clinical efficacy in the treatment of Alzheimer’s disease (AD). This study aims to explore the differences in therapeutic effects of DSS and its disassembled prescriptions, Suangan (SG) and Xingan (XG), in treating Alzheimer’s Disease and the mechanism of DSS recovering autophagy in AD.

**Methods:**

A network pharmacology strategy was employed to delineate the bioactive constituents, associated targets, and regulatory mechanisms of DSS in AD, encompassing in silico target forecasting, the generation and scrutiny of PPI networks, alongside GO and KEGG-based pathway elucidation. An AD mouse model, induced by intracerebroventricular injection of Aβ_1–42_, was used to evaluate the therapeutic effects of DSS and its disassembled prescriptions on AD. Cognitive function was evaluated using the Morris water maze. Expression levels of inflammatory cytokines were quantified via RT-qPCR and ELISA. Western blotting was used to detect the expression of proteins related to AD pathological markers and the AMPK/mTOR signaling pathway.

**Results:**

50 active compounds and 718 HUB genes were screened from relevant databases and literature. KEGG and GO analyses indicated that DSS’s potential mechanisms against AD involved the AMPK/mTOR signaling pathway and mitophagy. In vivo animal model, the results demonstrated that DSS, SG, and XG treatments improved cognitive function and ameliorated neuroinflammation in mice. Additionally, they alleviated the pathological changes of neuronal cells. These treatments also increased the protein level of PSD-95, and decreased levels of APP and p-Tau. Among them, DSS exhibited the best efficacy. Furthermore, DSS, SG, and XG upregulated the expression of LC3, Beclin1, and p-AMPK, while decreasing the expression of P62 and p-mTOR.

**Conclusions:**

DSS, SG, and XG were found to ameliorate AD-related pathological symptoms in Aβ_1−42_-injected mice, likely through the AMPK/mTOR autophagy signaling pathway.

**Supplementary Information:**

The online version contains supplementary material available at 10.1186/s12906-024-04588-x.

## Background

Alzheimer’s disease (AD) is a neurodegenerative disease that accounts for about 60–70% of dementia cases globally, with an increasing incidence that mainly affects the elderly and poses a serious risk to their health [1]. The pathogenesis of AD remains uncertain and is believed to result from various factors including amyloid beta (Aβ) plaque-associated neurodegeneration, neurofibrillary degeneration, synaptic dysfunction, neurotransmitter imbalance, neuroinflammation, autophagy damage, and intestinal flora imbalance, among others [2]. The medications used to treat AD only provide limited relief for certain symptoms and do not effectively prevent or reverse the progression of the disease, which imposes heavy economic burdens on individuals and society [3]. This underscores the urgent need for innovative and effective treatments for AD.

The pathogenesis of AD is complex, and the Aβ hypothesis is the mainstream theory of AD occurrence. The aggregation of Aβ outside neural cells forms senile plaques (amyloid plaques), which is one of the main pathological characteristics of AD. The excessive deposition of Aβ to form senile plaques can lead to nerve damage. With the increase in levels of inflammatory biomarkers such as interleukin beta 1 (IL-1β), interleukin-6 (IL-6), and tumor necrosis factor-alpha (TNF-α) produced by overactivation of glial cells, including microglia, in the brains of AD patients, as well as the discovery of AD risk genes associated with innate immune function, neuroinflammation has become another crucial factor in the onset of AD [4]. At the same time, Aβ deposition also accelerates microtubule-associated protein Tau fibril degeneration, further exacerbating the progression of AD [5]. Autophagy can remove damaged organelles and excess proteins, which is an important mechanism for maintaining normal cellular physiological functions. Studies [6–8] have shown that autophagy dysfunction is associated with AD, and restoring autophagy function can help improve neuroinflammation and reduce Aβ production, which helps in the treatment of AD.

Based on the complexity of the pathogenesis of AD, multi-target therapy with traditional Chinese medicine may be an effective treatment method. Danggui Shaoyao san (DSS) is an ancient Chinese medicinal formula that comes from the Synopsis of the Golden Chamber. DSS is composed of *Angelica Sinensis* (Oliv.) Diels., *Paeonia lactiflora* Pall., *Atractyolodes macrocephala* Koidz., *Poria cocos* (Schw.) Wolf., *Alisma orientale* (Sam.) Juzep., and *Ligusticum Chuanxiong* Hort. According to their properties, can be categorized into three taste properties: *Angelica Sinensis (Oliv.)* Diels. and Ligusticum *Chuanxiong* Hort. are pungent, *Paeonia lactiflora* Pall. is sour, and Atractyolodes *macrocephala* Koidz., *Poria cocos* (Schw.) Wolf., and *Alisma orientale* (Sam.) Juzep. are sweet.

The original formula is mainly used to treat women’s abdominal pain during pregnancy, and with the development of modern medicine, more and more studies have found that it has a good effect on the prevention and treatment of AD. When DSS is used as a compound prescription of Chinese medicine, high-throughput UPLC-Q-TOF-MS/MS analysis results showed that the components of DSS are complex and diverse [9]. Among them, 9 effective components have been screened out in the system network pharmacology, namely 3-butylphthalide, ligustilide, senkyunolide I, Z-butylidenephthalide, senkyunolide A, atractylenolide I, tetramethylpyrazine, ferulic acid, and gallic acid [10]. In clinical studies [11–14], DSS has been proven to improve patients’ cognitive function. What’s more, DSS has been proven to reduce amyloidosis and neurodegeneration by regulating lipid metabolism in AD mice [15]. Additionally, DSS has shown increased mitochondrial autophagy to remove damaged mitochondria via the PINK1-Parkin pathway, exhibited neuroprotective effects, and resisted neuronal death [16]. Moreover, it has been confirmed to reduce brain inflammation and improve central cholinergic nervous system dysfunction [17, 18]. Recent research [19] showed that DSS may alleviate cognitive disorders through the microbiome-gut-brain axis, which could lead to the creation of novel treatments for AD. Thus, this study aimed to further investigate the potential of the traditional Chinese medicine formula DSS as a multitarget therapy for AD, enriching the scientific basis for using DSS in the treatment of AD, considering its effectiveness and complexity.

## Materials and methods

### Screening of DSS chemical components and prediction of action targets

Utilizing the Traditional Chinese Medicine Systematic Pharmacology Database and Analysis Platform (TCMSP), a search was conducted for the chemical constituents of DSS with “Angelica sinensis,” “Paeonia lactiflora,” “Ligusticum chuanxiong,” “Atractylodes macrocephala,” “Poria cocos,” and “Alisma plantago-aquatica” as keywords. Preliminary screening for active compounds was performed based on two ADME properties: oral bioavailability (OB) ≥ 30% and drug-likeness (DL) ≥ 0.18. Furthermore, as supported by the available literature, additional compounds presented in DSS that potentially possessed therapeutic properties, but did not meet the aforementioned criteria of OB and DL, were included for comprehensive analysis. Structural data files for the aforementioned active compounds were obtained from the PubChem database (http://pubchem.ncbi.nlm.nih.gov/). The Swiss Target Prediction analysis platform (http://www.swisstargetprediction.ch/) was employed to predict the target proteins of the active compounds, and the target names were corrected by Uniport database (https://www.uniprot.org).

### AD-related target screening

Using “Alzheimer’s disease” as the keyword, potential targets associated with AD were retrieved from the OMIM database (http://www.omim.org), GeneCards database (http://www.genecards.org/), DRUGBANK database (https://go.drugbank.com/), and DisGeNET database (https://www.disgenet.org/). The disease targets were then entered into the UniProt database (https://www.uniprot.org) to standardize the gene nomenclature. After eliminating duplicates and targets that could not be standardized, the Alzheimer’s disease targets were identified. The targets of the active components of the medicine were combined with the disease targets and input into Venny 2.1 (https://bioinfogp.cnb.csic.es/tools/venny/index.html). The intersection of the two sets was taken as the key targets, that is, the potential therapeutic targets of DSS for the treatment of AD, and a Venn diagram was drawn.

### Construction and core target prediction of the DSS-AD PPI network

The key targets of DSS for the treatment of AD were uploaded to the STRING platform (https://string-db.org/) for Protein-Protein Interaction (PPI) analysis. The species is set to Homo Sapiens, the minimum required interaction score is set to > 0.7, and the other parameters are left unchanged by default, Isolated nodes in the network were removed to obtain the PPI network of DSS in the treatment of AD. The PPI network data were imported into Cytoscape 3.9.1 and further analyzed using the plugin CytoNCA. The connectivity (Degree), Betweenness Centrality (BC), and Closeness Centrality (CC) of the nodes in the PPI network were calculated, and the average values for Degree, BC, and CC were determined. Nodes and interactions that were simultaneously greater than the average values of Degree, BC, and CC were retained to construct the core target network of DSS in relation to AD.

### GO and KEGG pathway enrichment analysis

The key targets of DSS for the treatment of AD were imported into the DAVID database for analysis, which included the Gene Ontology (GO) analysis of biological processes (BP), molecular functions (MF), and cellular components (CC), as well as the Kyoto Encyclopedia of Genes and Genomes (KEGG) analysis. Ten species related to autophagy from each enrichment analysis result were taken for visualization.

### Molecular docking

The mol2 format file of the active ingredient was downloaded via TCMSP, and the 3D crystal structures of AMPK and mTOR were obtained on the PDB Protein Data Bank (https://www.rcsb.org/). Subsequently, the proteins and the active ingredients were imported into the AutoDock platform for docking analysis.

### Antibodies and reagents

LC3B (ab192890), Beclin1 (ab207612),PSD-95 (ab238135) and IBA1 (ab178846) were purchased from Abcam (Cambridge, MA, USA). P62 (T55546S), p-AMPK (T55608S), and p-mTOR (T565571S) were purchased from Abmart Shanghai Co., Ltd. (Shanghai, China). APP (AF6084), GADPH (T0004) was purchased from Affinity Biosciences LTD (Jiangsu, China), p-Tau (WL03540) was purchased from WanLeiBio (Shenyang, China), IL-1β, IL-6, and TNF-α ELISA (enzyme-linked immunosorbent assay) kits were purchased from UpingBio (Shenzhen, China). EZ-press RNA Purification Kit, Color Reverse Transcription Kit, and 2Color SYBR Green qPCR Master Mix (ROX2 plus) were purchased from EZE Science Biotechnology (Roseville, NM, USA). Human Aβ_1–42_ peptide (Catalog: AG968) was purchased from Sigma-Aldrich Chemical Co. (St Louis, MO, USA).

### Preparation of DSS, SG, and XG

DSS is composed of six raw herbs, including *Angelica Sinensis* (Oliv.) Diels. (Batch numbers:221,103,461), *Paeonia lactiflora* Pall. (Batch numbers:221,100,229), *Atractyolodes macrocephala* Koidz. (Batch numbers:221,260,491), *Poria cocos* (Schw.) Wolf. (Batch numbers:221,202,451), *Alisma orientale* (Sam.) Juzep. (Batch numbers:221,000,671), and *Ligusticum Chuanxiong* Hort. (Batch numbers:221,101,331) were sourced from Kangmei Pharmaceutical Co., Ltd. (Guangdong, P.R. China) and they were combined in a ratio of 3: 16: 4: 4: 8: 8. The Suangan (SG) is composed of *Paeonia lactiflora* Pall., *Atractyolodes macrocephala* Koidz., *Poria cocos* (Schw.) Wolf., *Alisma orientale* (Sam.) Juzep. and they were combined in a ratio of 16: 4: 4: 8. The Xingan (XG) is composed of *Angelica Sinensis* (Oliv.) Diels., *Ligusticum Chuanxiong* Hort., *Atractyolodes macrocephala* Koidz., *Poria cocos* (Schw.) Wolf., *Alisma orientale* (Sam.) Juzep. and they were combined in a ratio of 3: 8: 4: 4: 8. Subsequently, the mixture of dried herbs was briefly immersed in distilled water at a volume-to-weight ratio of 8:1 for one hour, followed by decoction for another hour. The resulting filtrate was collected, and the residue was subjected to a second round of decoction using distilled water at a volume-to-weight ratio of 6:1 for one hour. The filtrates obtained from both decoction rounds were combined and then concentrated.

### Animals and drug treatment

Eight-week-old male and female C57BL/6J mice were purchased from Guangzhou Ruige Biological Technology Co., Ltd. The experimental animals were housed in a specific pathogen-free (SPF) room with a controlled temperature of 25 ℃, a relative humidity ranging from 50 to 70%, and a 12-hour light/dark cycle. They were provided with free access to water and food. The experimental protocols were approved by the Animal Experimentation Committee at Guangzhou University of Chinese Medicine and conducted following the National Research Council Guide for Care and Use of Laboratory Animals (approval number: ZYD-2022-267).

### Groups and drug administration

After one week of acclimation, all animals were randomly divided into six groups, each consisting of eight mice (four males and four females): the sham group, model group, DSS group, SG group, XG group, and Donepezil group. The Donepezil group was used as a positive control and administered at a dosage of 3 mg/kg/day.

Before the surgical procedure, Aβ_1−42_ was dissolved in the sterile saline solution to achieve a concentration of 1 µg/1µl. Subsequently, the solution underwent aggregation by incubating it at 37 °C for 7 days [20]. Based on the mouse brain atlas, after anesthetizing with 0.25 g/kg tribromoethanol, all mice except those in the sham group were intracerebroventricularly (ICV) injected with aggregated Aβ_1−42_ peptide on each side, totaling 5 µl per injection site. The injection coordinates were as follows: AP (anteroposterior): -1.94 mm; ML (mediolateral): ±l.4 mm; DL (dorsoventral): -2.2 mm. In contrast, the sham group received intracerebroventricular injections of physiological saline at the same coordinates. To minimize backflow, the needle was kept in the injection site for 5 min [21].

On the third day after the injection, the treatment regimen was administered based on previous studies [22]. The experimental groups were given DSS (6.4 g/kg/day), SG (4.8 g/kg/day), XG (4.0 g/kg/day), and Donepezil (3 mg/kg/day) daily for 28 consecutive days through oral gavage. In contrast, the control and model groups received the same volume of saline solution. The dosages for the SG and XG groups were adjusted proportionally, referring to the recommended amounts in the book " Synopsis of the Golden Chamber “.

### Morris water maze test (MWM test)

The MWM test was performed three weeks following the administration of the drug. It involves a circular pool measuring 1600 cm in diameter and 50 cm in height. The pool is divided into four equal quadrants. The pool is filled with opaque water, and in the designated quadrant, there is a platform with a diameter of 10 cm that is not easily noticeable underwater. The platform is situated 1.0 cm below the water surface.

Animals were randomly released from different quadrants of the pool to train them to locate the hidden platform after a day of acclimatizing to the pool. They underwent this training three times a day for five days, with a 20-minute interval between trials. If the mice failed to find the platform within 60 s, their latency was recorded as 60 s, and they were then guided to the platform location for 10 s. The average escape latency of each animal was recorded daily.

**Fig. 1:**
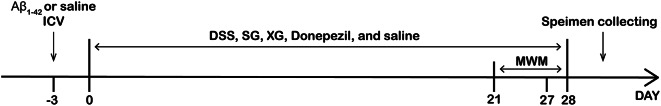
Experimental flow chart for the Aβ_1−42_ (ICV) model, behavioral studies, and biochemical and molecular analysis

On the sixth day, the mice underwent a probe trial session in which the platform was removed from the pool. The mice were released at the opposite point of the platform’s original position and allowed to swim for 60 s to search for the platform. The time spent in the target quadrant and the number of times each mouse crossed the platform were recorded and analyzed. The experimental design of the conducted studies is delineated in Fig. 1.

### Specimen collection and storage

On the following day after the MWM experiment, mice were euthanized by cervical dislocation after being deeply anesthetized with 0.25 g/kg of tribromoethanol, and then their brains were dissected and separated into left and right hemispheres. The left hemisphere was histologically examined and preserved in 4% paraformaldehyde. The cortex and hippocampus of the right hemisphere were isolated and rapidly frozen in liquid nitrogen before being transferred to a refrigerator set at -80 ℃ for molecular biological analysis.

### Real-time quantitative PCR (RT-qPCR)

Tissues were homogenized following the instructions of the manufacturer. Total RNA was extracted from the tissues using the EZ-press RNA Purification Kit reagent (EZBioscience, Roseville, MN, USA). mRNA was reverse transcribed into Complementary DNA (cDNA) using a cDNA synthesis kit (EZBioscience, Roseville, NM, USA). Subsequently, the cDNA was then combined with specific primers according to the 2x Color SYBR Green qPCR Master Mix (EZBioscience, Roseville, NM, USA). The relative expression of the target genes was determined by qPCR, with GADPH serving as the internal control. The relative expression levels of mRNA were calculated using the 2^-ΔΔCT^ method.

The primer sequences can be found in the Table 1.

**Table 1:** The primer sequences for each gene

Gene	Forward(5′-3′)	Reverse(5′-3′)
IL-1β	CTCGCAGCAGCACATCAACAAG	CCACGGGAAAGACACAGGTAGC
IL-6	TTCTTGGGACTGATGCTGGTGAC	GTGGTATCCTCTGTGAAGTCTCCTC
TNF-α	ACGCTCTTCTGTCTACTGAACTTCG	TGGTTTGTGAGTGTGAGGGTCTG
GAPDH	AGAAGGTGGTGAAGCAGGCATC	CGAAGGTGGAAGAGTGGGAGTTG

### Enzyme-linked immunosorbent assay (ELISA)

The levels of IL1β, IL6, and TNF-α were measured utilizing the ELISA assay kits (UpingBio, Shenzhen, China) according to the manufacturer’s directions with a multifunctional microplate reader.

### Western blot

Brain tissues were briefly lysed in RIPA lysis buffer supplemented with protease and phosphatase inhibitors cocktail. The supernatant was collected, and BCA assays determined the protein concentration. Protein (40 µg) was resolved by 10-12% SDS-PAGE gel electrophoresis and subsequently transferred to polyvinylidene difluoride membranes. After blocking with 5% skim milk for 1 h at room temperature, the membranes were incubated with corresponding primary antibodies: PSD-95, APP, p-mTOR, p-AMPK, P62, Beclin1, LC3B, and GADPH overnight at 4 ℃. On the following day, after three incubations in TBST of 5 min each, the membranes were incubated with horseradish peroxidase (HRP)-conjugated anti-mouse (1: 5000) or Goat anti-rabbit IgG (1: 5000) for 1 h at room temperature. Finally, an ultrasensitive ECL chemiluminescent solution (BL520A, Biosharp, China) was applied, and images were captured by the Chemiluminescence image analysis system (Shanghai Tanon, China). The densitometry of the bands was visualized using ImageJ software.

### Hematoxylin-eosin (HE) staining

After being placed at a constant temperature of 65 °C for 1.5 h, brain paraffin sections were washed and dehydrated in alcohol, deparaffinized in xylene, and stained with hematoxylin-eosin for observation of cell morphology.

### Immunohistochemistry

Brain paraffin sections were kept at a constant temperature of 65 °C for 1.5 h. Subsequently, the brain sections were dewaxed and rehydrated using various concentrations of ethanol. Antigenic repair was performed using citric acid. Endogenous peroxidase activity was blocked using blocking agents (ZSB-BIO, Beijing, China). The sections were then blocked with 3% bovine serum albumin for 1 h before being incubated overnight at 4 ℃ with IBA1 (diluted 1:1000). The following day, secondary antibodies (Enhanced enzyme-labeled goat anti-rabbit IgG polymer, ZSB-BIO, Beijing, China) were added. The sections were covered with the DAB Detection Kit (ZSB-BIO, Beijing, China) and counterstained with hematoxylin. Finally, the sections were photographed under a microscope, and the IOD value was analyzed using Image-Pro Plus.

### Statistical analysis

The data are presented as the mean ± SEM. Statistic Package for Social Science (SPSS, v.20.0) statistical software was used for the data analysis. To analyze the escape latency data in the Morris water maze, repeated measures analysis of variance (ANOVA) was employed. For the analysis of other data, a one-way ANOVA followed by post hoc LSD or Dunnett’s T3 test was used. A statistical significance level of *P* < 0.05 was considered.

## Results

### DSS effective component screening and action target prediction results

A total of 50 active components were identified, including 3 from *Angelica Sinensis* (Oliv.) Diels., 10 from *Paeonia lactiflora* Pall., 7 from *Ligusticum Chuanxiong* Hort., 8 from *Atractyloides macrocephala* Koidz., 15 from *Poria cocos* (Schw.) Wolf., and 10 from *Alisma orientale* (Sam.) Juzep. Their basic information can be found in Table S1. Utilizing the Swiss Target Prediction analysis platform, 718 potential targets for the 50 active components were collected.

### Disease target and network construction

**Fig. 2:**
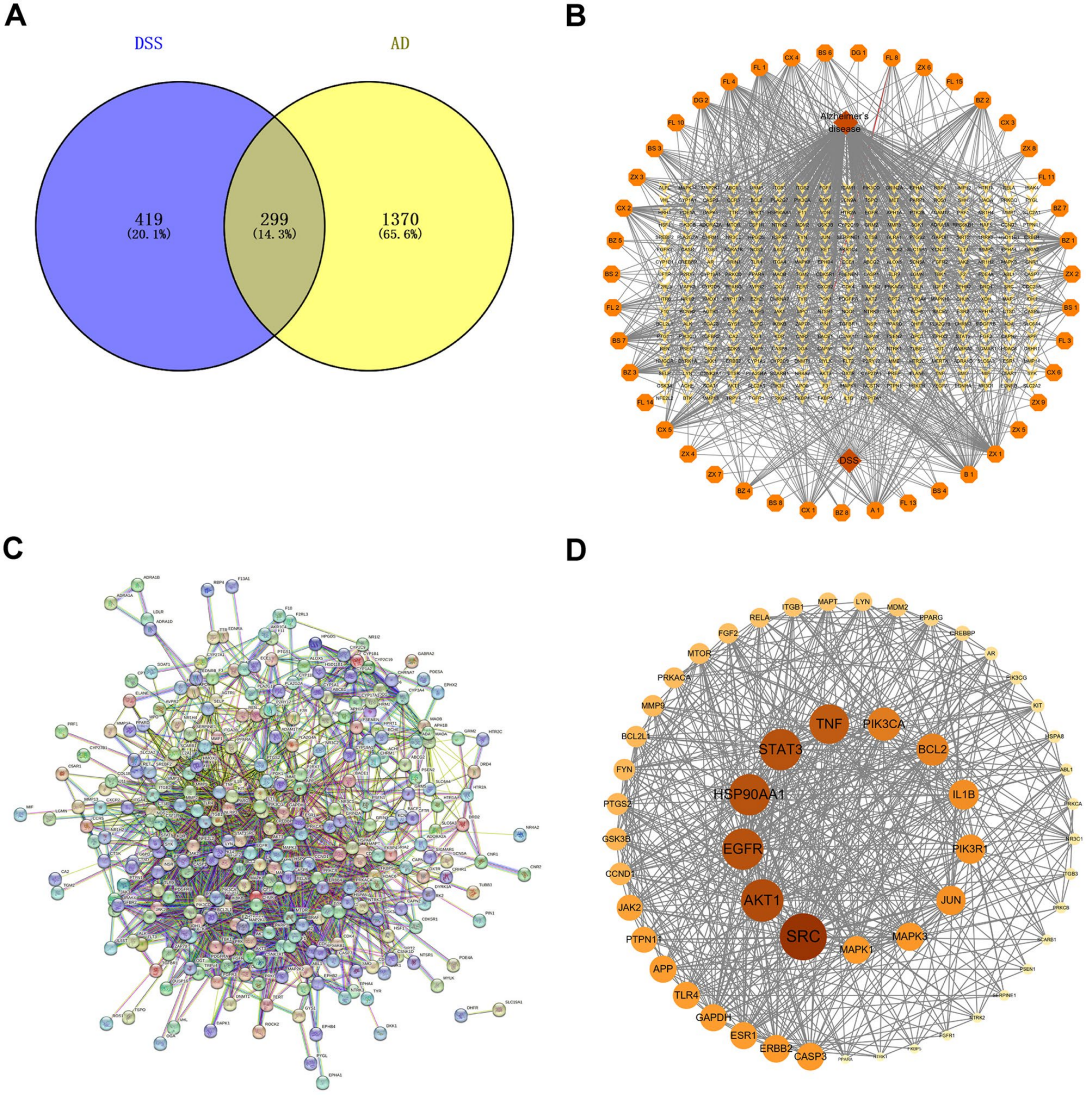
Disease target and network construction. (**A**) Venn diagram of DSS and AD. (**B**) DSS medicine-active ingredients-target-disease network. (**C**) Cross-PPI networks. (**D**) PPI network core screening

A total of 14,316; 144; 1,848; and 49 targets were collected from the Gene Cards, OMIM, DisGeNET, and Drug Bank databases, respectively. After screening with a score of ≥ 54 from Gene Cards, 904 targets were retained. From DisGeNET, after screening with a score of ≥ 0.05, 904 targets were also retained. After merging and removing duplicates, a final total of 1,669 targets were obtained. The intersection of the screened DSS active component targets and AD targets was taken, and a Venn diagram was drawn using Venny 2.1 (Fig. 2A), resulting in 299 common targets between DSS and AD and mapping the DSS medicine-active ingredients-target-disease network (Fig. 2B). Subsequently, the intersection targets were submitted to the STRING v11.0 platform, yielding the PPI network of DSS targets (Fig. 2C). The obtained PPI network relationships were imported into Cytoscape 3.9.1 software, and the CytoNCA plugin was utilized to calculate the Degree, Betweenness Centrality (BC), and Closeness Centrality (CC) values for each node in the PPI network. The average values for Degree, BC, and CC were computed, and nodes and interactions that were simultaneously greater than the average values for Degree, BC, and CC were retained. This process led to the construction of the core target network for DSS in relation to AD (Fig. 2D), with information on the top ten core targets presented in Table 2.

**Table 2:** Potential targets of DSS for the treatment of AD

No.	Symbol	Degree	Betweenness	Closeness
1	SRC	82	7519.6406	0.53962266
2	AKT1	74	4065.4417	0.517179
3	HSP90AA1	72	4448.968	0.5061947
4	EGFR	72	3652.1936	0.5247706
5	STAT3	72	3286.0574	0.513465
6	TNF	69	6982.3545	0.50352114
7	PIK3CA	58	1872.9424	0.46129033
8	BCL2	56	2395.5217	0.48639455
9	IL1B	52	2508.5764	0.48474577
10	PIK3R1	51	1046.3677	0.4532488

### GO and KEGG analysis

**Fig. 3:**
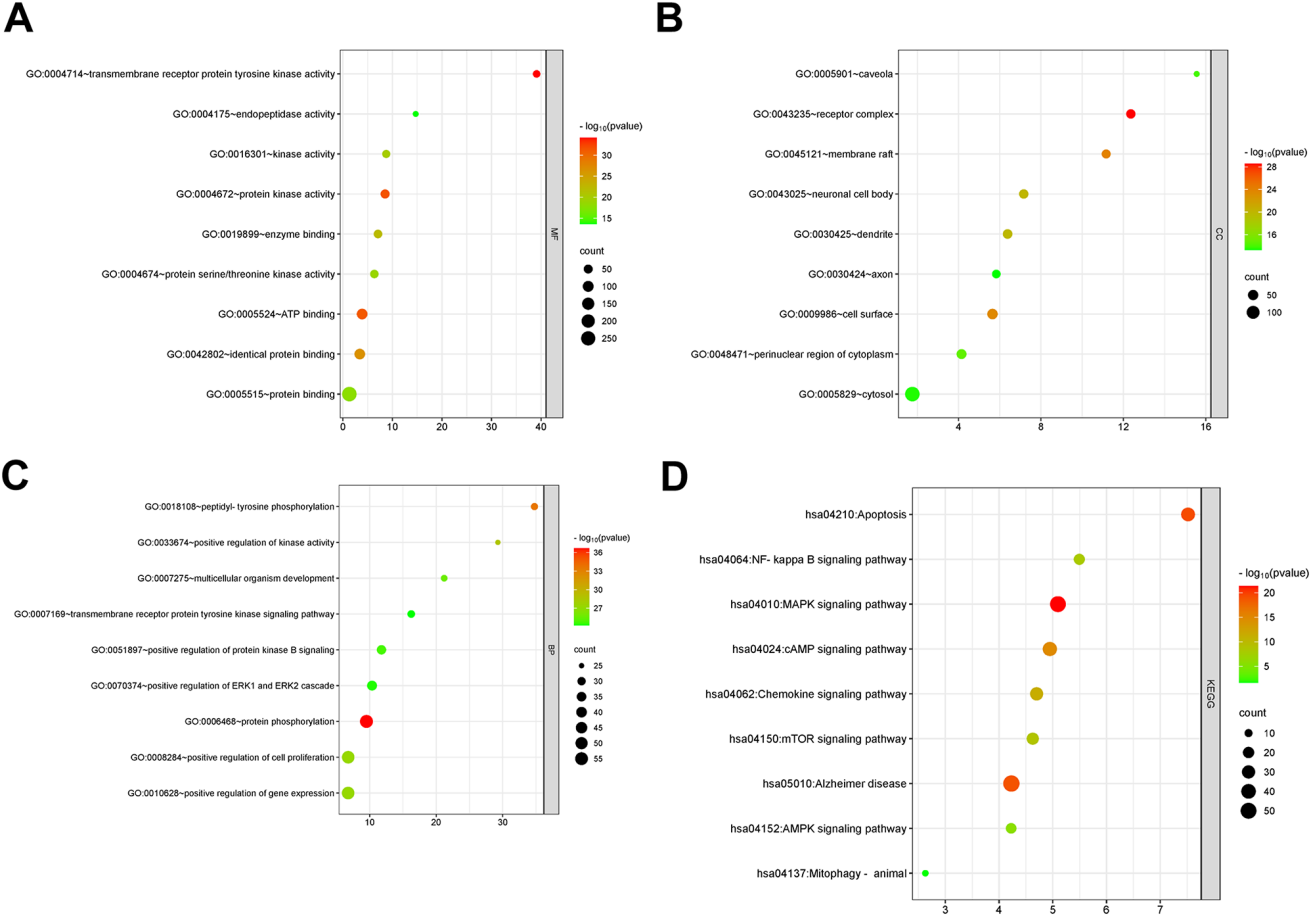
GO and KEGG analysis. (**A**) molecular function. (**B**) cellular components. (**C**) biological processes. (**D**)Results of KEGG enrichment

GO functional enrichment analysis and KEGG pathway analysis were performed on 299 intersecting targets using the DAVID database. A total of 1,186 entries with *p* < 0.05 were obtained for GO function enrichment analysis, including and 184 entries (Fig. 3A) for molecular function (MF), 115 entries (Fig. 3B) for cellular component (CC), 887 entries (Fig. 3C) for biological process (BP), and the results of KEGG pathway analysis showed that a total of 179 pathways (Fig. 3D) with *P* < 0.05 were obtained. 10 results related to autophagy were extracted from each enrichment analysis result for visualization.

### Molecular docking

KEGG and GO analyses indicated that the potential mechanisms of DSS anti-AD effects involved the AMPK/mTOR signaling pathway and mitochondrial phagocytosis. We selected the AMPK/mTOR signaling pathway for subsequent validation. The main active components in DSS were selected for molecular docking with AMPK and mTOR and the binding energies were calculated, and the specific results are listed below (Table 3). It is generally believed that a binding energy <-4.25 suggests low ligand-receptor binding activity, <-5.0 moderate binding activity, and <-7.0 high binding activity.

**Table 3:** Binding abilities between drug components of DSS and target proteins

Protein	PDBID	Ligand	Binding Afnity	Protein	PDBID	Ligand	Binding Afnity
AMPK	4RED	paeoniflorin	-7.9	mTOR	5WBH	paeoniflorin	-7.8
AMPK	4RED	ferulic acid	-6	mTOR	5WBH	ferulic acid	-6.5
AMPK	4RED	atractylenolide III	-7	mTOR	5WBH	atractylenolide III	-7.2
AMPK	4RED	12-senecioyl-2E,8E,10E-atractylentriol	-5.7	mTOR	5WBH	12-senecioyl-2E,8E,10E-atractylentriol	-6.8
AMPK	4RED	Alisol B	-7.7	mTOR	5WBH	Alisol B	-7.9
AMPK	4RED	sitosterol	-6.6	mTOR	5WBH	sitosterol	-6.5
AMPK	4RED	Cerevisterol	-7.4	mTOR	5WBH	Cerevisterol	-7.4
AMPK	4RED	14-acetyl-12-senecioyl-2E,8E,10E-atractylentriol	-5.5	mTOR	5WBH	14-acetyl-12-senecioyl-2E,8E,10E-atractylentriol	-6.9
AMPK	4RED	kaempferol	-7.3	mTOR	5WBH	kaempferol	-9.3
AMPK	4RED	Myricanone	-7.5	mTOR	5WBH	Myricanone	-7.9
AMPK	4RED	wallichilide	-7.2	mTOR	5WBH	wallichilide	-7.4
AMPK	4RED	beta-sitosterol	-7.4	mTOR	5WBH	beta-sitosterol	-8.6
AMPK	4RED	paeoniflorin	-7.9	mTOR	5WBH	paeoniflorin	-7.8
AMPK	4RED	ferulic acid	-6	mTOR	5WBH	ferulic acid	-6.5
AMPK	4RED	atractylenolide III	-7	mTOR	5WBH	atractylenolide III	-7.2
AMPK	4RED	(2R)-2-[(3 S,5R,10 S,13R,14R,16R,17R)-3,16-dihydroxy-4,4,10,13,14-pentamethyl-2,3,5,6,12,15,16,17-octahydro-1 H-cyclopenta[a]phenanthren-17-yl]-6-methylhept-5-enoic acid	-7.9	mTOR	5WBH	(2R)-2-[(3 S,5R,10 S,13R,14R,16R,17R)-3,16-dihydroxy-4,4,10,13,14-pentamethyl-2,3,5,6,12,15,16,17-octahydro-1 H-cyclopenta[a]phenanthren-17-yl]-6-methylhept-5-enoic acid	-8

### DSS, SG, and XG improve the learning and memory function in Aβ_1−42_-injected mice

**Fig. 4:**
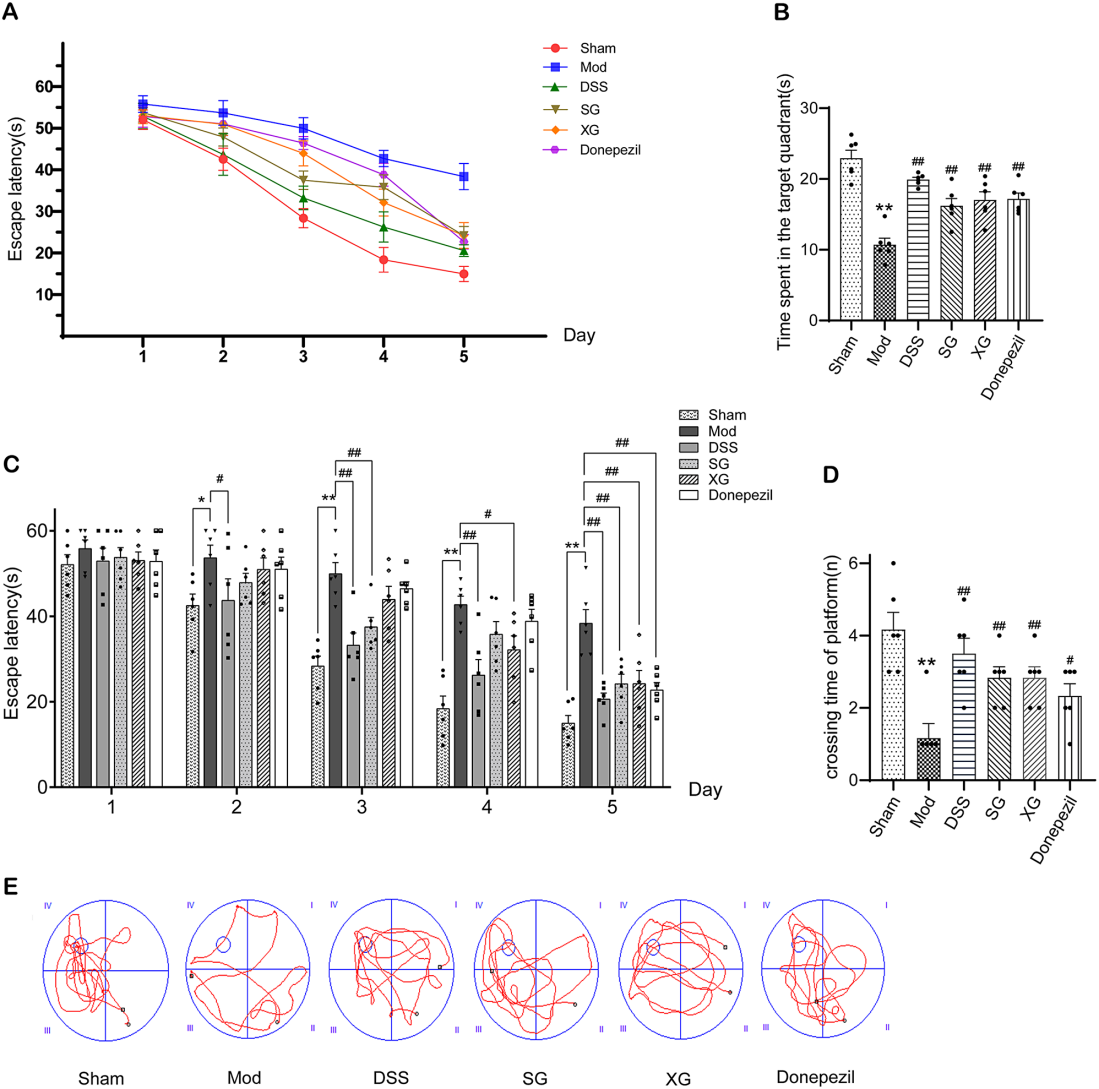
Effects of DSS, SG, and XG on MWM test in Aβ_1−42_-injected mice. (**A**) An escape incubation period of 5 consecutive days. (**B**)Time spent in the target quadrant. (**C**) Daily escape latency. (**D**) Crossing time of platform location. (**E**) Movement trajectory of mice. Data are presented as mean ± SEM, (*n* = 6);^*^P < 0.05, ^**^P < 0.01, compared with the Sham group. ^#^*P* < 0.05, ^##^*P* < 0.01, compared with the Model group

The escape latency of the mice decreased progressively during the 5-day location navigation test, with or without drug treatment (Fig. 4A). The escape latency of mice in each group was assessed daily (Fig. 4C). From the second day onwards, the model mice exhibited the longest time to locate the platform, whereas the DSS, SG, and XG significantly reduced the time taken to find the platform. Additionally, Donepezil also significantly shortened the time taken to find the platform. In the spatial exploration experiment, the model group exhibited the least amount of time spent in the target quadrant and the fewest platform crossings (Fig. 4B, D). Compared to the model group, the DSS, SG, XG, and Donepezil groups all increased the time spent in the target quadrant and the number of platform crossings (*P < *0.05 or *P* < 0.01), with the DSS demonstrating the highest time spent in the target quadrant and the greatest number of platform crossings. The typical swimming paths are depicted in Fig. 4E. Remarkably, DSS showed the most significant efficacy among the three treatment groups (SG, XG, and DSS).

### DSS, SG, and XG improved neuroinflammation in Aβ_1−42_-injected mice

**Fig. 5:**
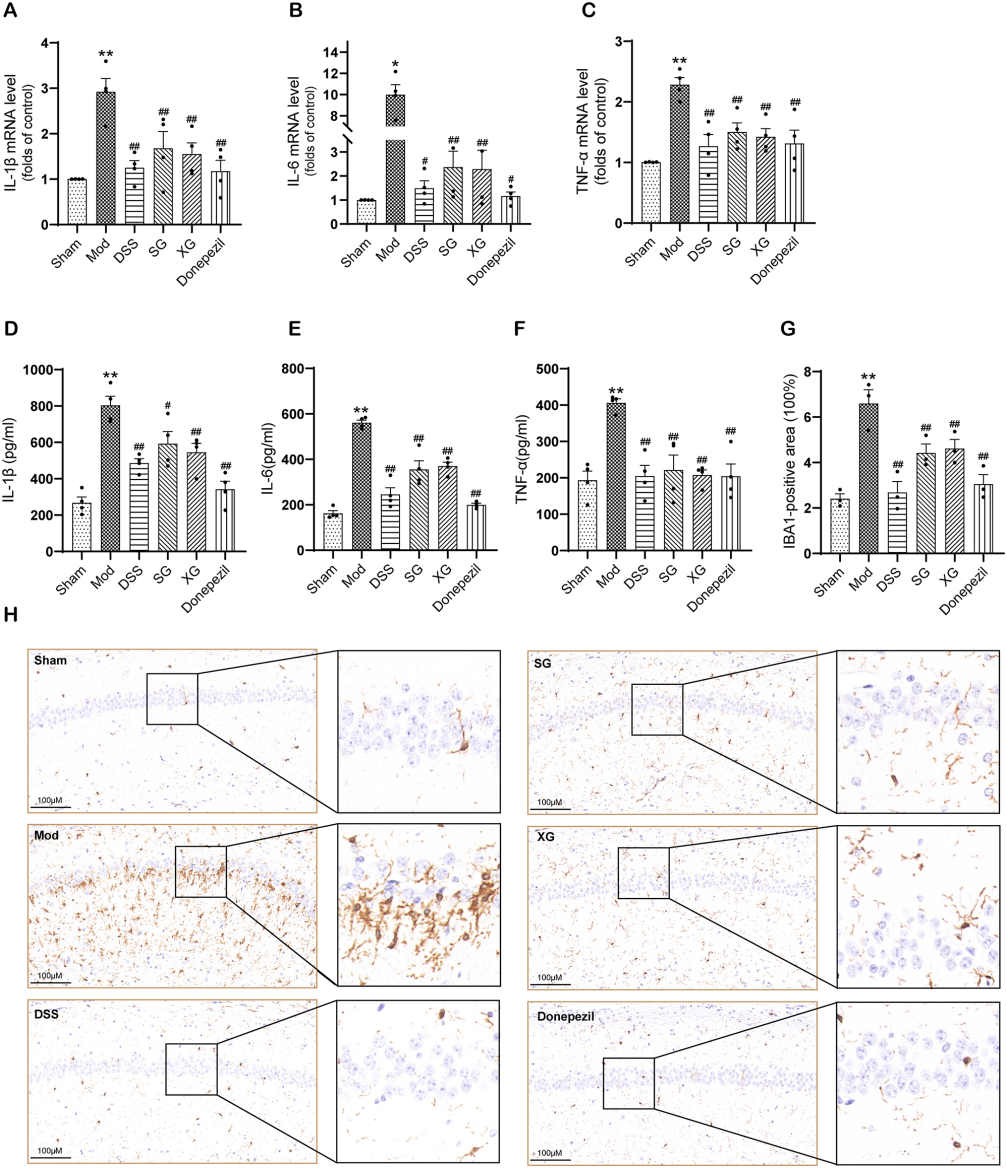
DSS, SG, and XG reduced the expression of inflammatory factors in Aβ_1−42_-injected Mice. (**A**–**C**) mRNA expression levels of IL-1β, IL-6, and TNF-α determined by RT-qPCR. (**D**–**F**) The protein expression levels of IL-1β, IL-6, and TNF-α determined by ELISA. (**G**–**H**) Representative image and expression of IBA1. Data are presented as mean ± SEM, (*n* = 3 ∼ 4); ^*^P < 0.05, ^**^P < 0.01compared with the Sham group. ^#^*P* < 0.05, ^##^*P* < 0.01 compared with the Model group

To investigate the effects of DSS, SG, and XG on the expression of inflammatory factors, we used RT-qPCR and ELISA kits to evaluate the levels of IL-1β, IL-6, and TNF-α in the brain tissue. The RT-qPCR results (Fig. 5A-C) demonstrated that compared with the sham group, the mRNA levels of IL-1β, IL-6, and TNF-α in the model group were increased, while DSS, SG, XG, and Donepezil exhibited the ability to decrease the mRNA expression levels of IL-1β, IL-6, and TNF-α when compared to the model group ( *P*< 0.05 or *P* < 0.01). Similarly, the ELISA results (Fig. 5D-F) indicated that DSS, SG, XG, and Donepezil were effective in reducing protein levels of IL-1β, IL-6, and TNF-α compared to the model group (*P* < 0.05 or *P* < 0.01). In addition, the activation of microglia is associated with the release of inflammatory factors. Immunohistochemical results (Fig. 5G-H) showed that, compared to the model group, DSS, SG, XG, and Donepezil could inhibit IBA1 expression (*P* < 0.01). Therefore, these findings suggested that treatment with these drugs could effectively ameliorate neuroinflammation in Aβ_1−42_-injected mice. Among them, the effect of DSS was more significant.

### DSS, SG, and XG improve neuronal degeneration in Aβ_1−42_-injected mice

**Fig. 6:**
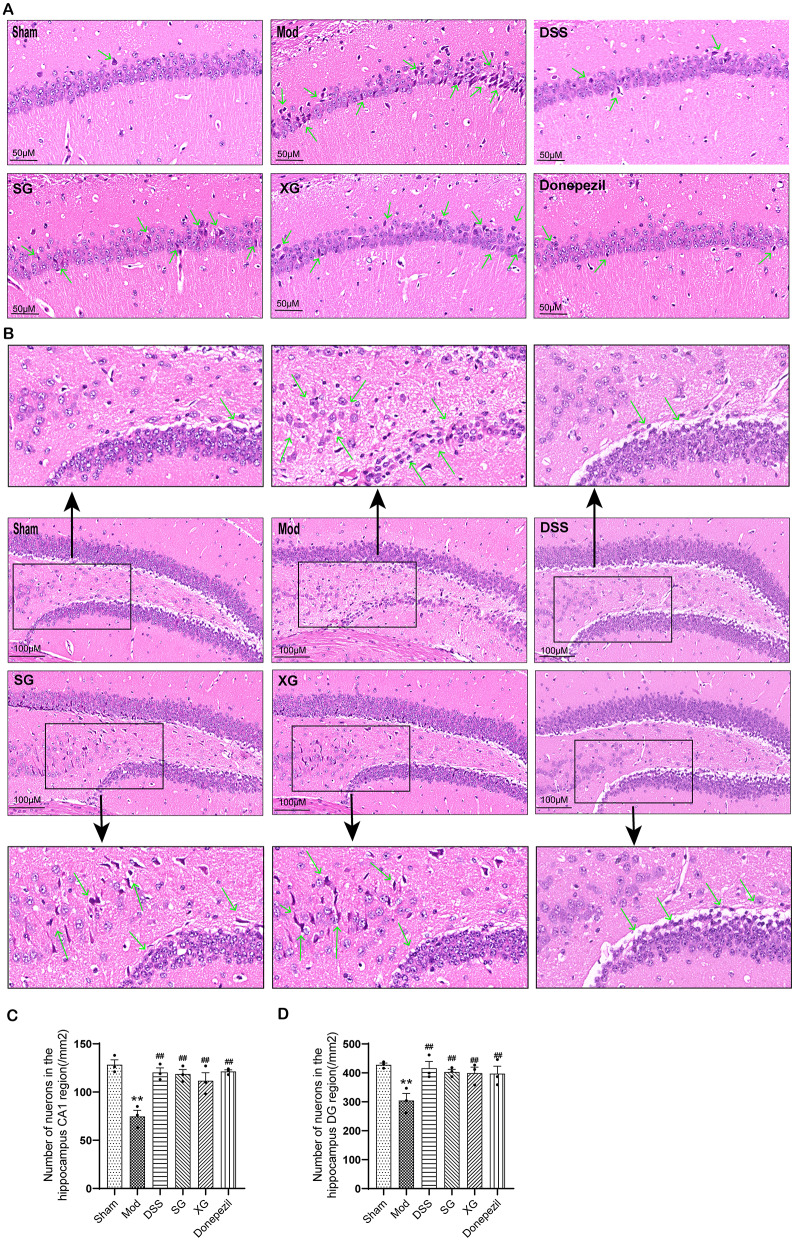
(**A**) HE staining of the hippocampal CA1 region. (**B**) HE staining of the hippocampal DG region. (**C**) Number of neuronal cells in hippocampus CA1. (**D**) Number of neuronal cells in hippocampus DG. Data are presented as mean ± SEM, (*n* = 3); ^*^P < 0.05, ^**^P < 0.01compared with the Sham group. ^#^*P* < 0.05, ^##^*P* < 0.01 compared with the Model group

It is widely accepted that the hippocampus plays an essential role in memory, learning, and cognitive abilities. Research has demonstrated that the CA1 region of the hippocampus is closely associated with cognitive function and is vulnerable to brain damage [23]. According to the HE staining results depicted in Fig. 6A, it was evident that the sham group exhibited intact cell morphology with neurons neatly arranged. Conversely, the neurons in the model group displayed irregular arrangement, loose organization, and noticeable neuron loss. After treatment, the number of deeply stained nuclei in the mice across all groups decreased, indicating a mitigation of neuronal cell morphological damage. The arrangement of neurons became more orderly, and there was an increase in cell quantity (Fig. 6C).

The results of the dentate gyrus (DG) region in the hippocampus as shown in Fig. 6B, the sham group exhibited normal cellular morphology with cells that were neatly arranged and displayed typical histological features of healthy neurons. In contrast, the model group showed a reduction in cell count, disordered cellular arrangement, and pyknotic deformation indicative of cellular degeneration. In the treatment groups, there was a noticeable increase in cell number, and the cellular morphology appeared to be more regular. As depicted in Fig. 6D, the quantitative outcomes demonstrated a coherent pattern.

### DSS, SG, and XG increased the expression of PSD-95 and decreased the expression of APP and p-Tau in Aβ_1−42_-injected mice

**Fig. 7:**
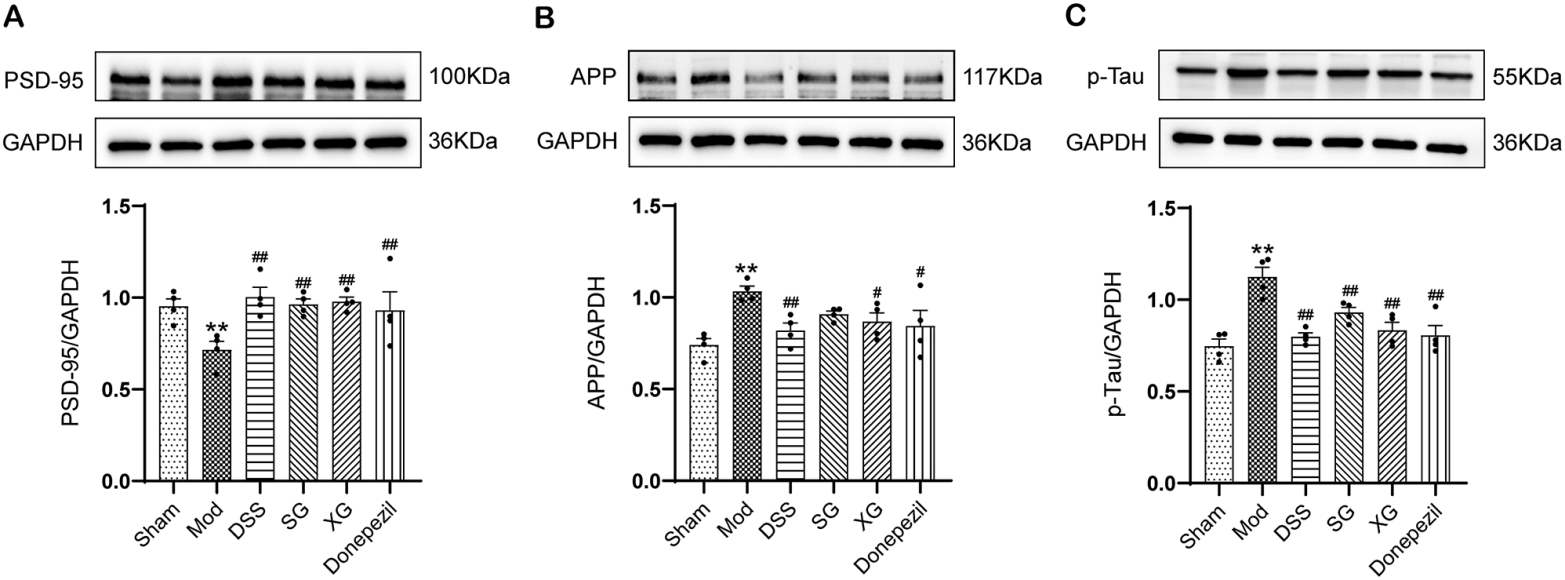
DSS, SG, and XG increased the expression of PSD-95 and decreased the expression of APP and p-Tau in Aβ_1−42_-injected Mice. (**A**–**C**) PSD-95, APP, and p-Tau were detected by western blot. Full-length blots are presented in Supplementary Fig. 1. Data are presented as mean ± SEM, (*n* = 4); ^*^P < 0.05, ^**^P < 0.01compared with the Sham group. ^#^*P* < 0.05, ^##^*P* < 0.01 compared with the Model group

The expression of postsynaptic density protein 95 (PSD-95), amyloid precursor protein (APP), and hyperphosphorylated microtubule-associated protein tau (p-Tau) was detected through Western blot analysis (Fig. 7). The result (Fig. 7A) demonstrated that PSD-95 expression was decreased in the model group compared to the sham group while it was restored in all treatment groups after administration (*P* < 0.01). Conversely, the model group exhibited increased expression of APP and p-Tau (Fig. 7B-C) compared to the sham group, which decreased in all treatment groups after administration (*P* < 0.05 or *P* < 0.01). However, there was no significant difference in SG’s APP expression (*P* > 0.05).

### Effects of DSS, SG, and XG on autophagy and AMPK/mTOR signaling pathway in Aβ_1−42_-injected mice

**Fig. 8:**
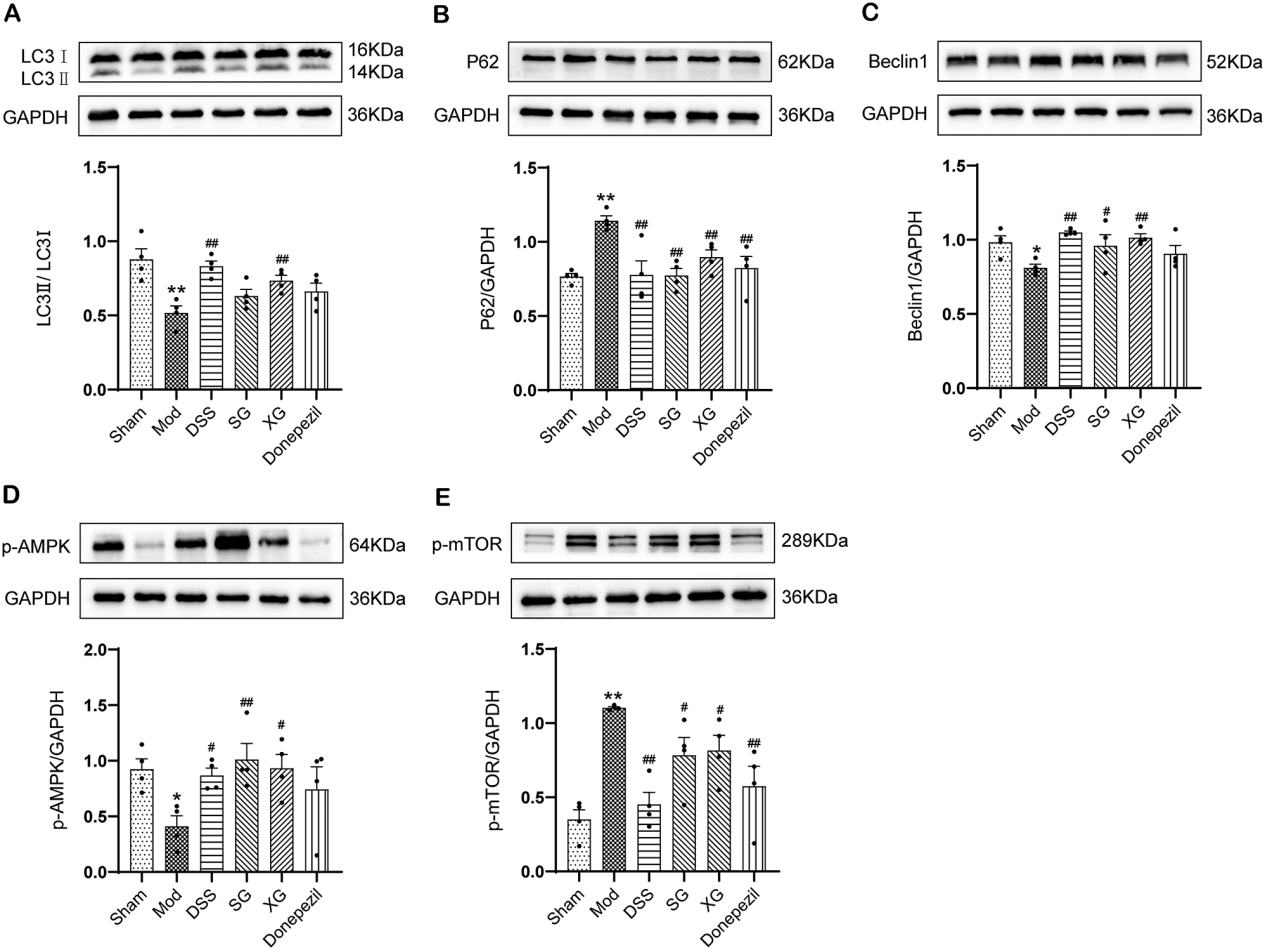
DSS, SG, and XG activate autophagy and AMPK/mTOR signaling pathway in Aβ_1−42_-injected Mice. (**A**–**E**) The expression of autophagy proteins LC3, P62, Beclin1, p-AMPK, and p-mTOR was detected using Western blot analysis. Full-length blots are presented in Supplementary Figs. 2–3. Data are presented as mean ± SEM, (*n* = 4); ^*^P < 0.05, ^**^P < 0.01compared with the Sham group. ^#^*P* < 0.05, ^##^*P* < 0.01 compared with the Model group

Western blot was used to analyze the expression levels of microtubule-associated protein 1 light chain 3 (LC3), P62, and Beclin1 to determine whether the treatment effects of DSS, SG, and XG on AD were associated with autophagy (Fig. 8A-C). The results showed that in the model group, the expression of LC3 and Beclin1 decreased compared to the sham group, but had exhibited recovery after drug administration. Only DSS and XG, however, compared with the model group, displayed statistically significant differences in the LC3 expression analysis (*P* < 0.01). In addition, compared to the sham group, the model group exhibited increased expression of P62 (*P* < 0.01). After drug administration, the P62 levels decreased in all groups (*P* < 0.01). These results suggested that DSS, SG, and XG treatment for AD may be associated with the restoration of Aβ-induced autophagy inhibition.

Next, to further determine whether DSS, SG, and XG had activated the adenosine 5′-monophosphate (AMP)-activated protein kinase (AMPK)/mammalian target of rapamycin (mTOR) signaling pathway, we adopted Western blot to detect the expression levels of Phosphorylated AMPK (p-AMPK) and Phosphorylated mTOR (p-mTOR) in each group (Fig. 8D-E). The results showed that after Aβ induction, the expression of p-AMPK decreased in the model group, while the expression of p-mTOR increased (*P* < 0.05 or *P* < 0.01). Compared to the model group, the DSS, SG, and XG showed increased expression of p-AMPK, and decreased expression of p-mTOR (*P* < 0.05 or *P* < 0.01).

## Discussion

AD is a neurodegenerative disorder characterized by the progressive deterioration of cognitive functions over time, with significant impacts on learning and memory capability [1].The MWM, established by Morris in 1981, is designed to assess the spatial learning and memory capabilities of experimental animals in perceiving location and direction [24]. After Aβ injection, mice showed increased escape latency, which was reduced by treatment with DSS, SG, XG, and Donepezil. This suggests improved learning in treated mice, with the DSS group showing the most significant early improvement. Spatial exploration tests revealed that treated mice remembered the platform location better than the model group, with the DSS demonstrating the best memory retention, indicating its potential in enhancing learning and memory in AD model mice.

The interaction between Aβ, p-Tau, and neuroinflammation is a characteristic pathogenic feature of AD that accelerates the disease’s progression [5, 25]. According to reports [20, 26], intracerebral injection of Aβ in mice can induce neuroinflammation and p-Tau. Neuroinflammation refers to the inflammatory responses within the nervous system. When neural tissues are damaged, infected, or subjected to other stimuli, immune cells release inflammatory mediators, causing inflammatory reactions in the neural tissue [4]. Common inflammatory cytokines, such as IL-1β, IL-6, and TNF-α, are essential for the body’s immune system’s regulation and inflammatory response. When the insoluble Aβ penetrates the brain, it triggers glial cells to become over-activated, which releases excess inflammatory cytokines and causes neuroinflammation. This neuroinflammation, in turn, induces synaptic degeneration, neurodegeneration, exacerbation of diverse brain lesions, and an elevated accumulation of insoluble Aβ and p-Tau, thereby instigating a detrimentalcycle [27].

Microglia are the primary innate immune cells in the brain, and their activation is, to a certain extent, a defensive response to inflammatory stimuli. The purpose of this activation is to eliminate pathogens, maintain tissue homeostasis, and facilitate the repair process [28]. However, excessive or sustained activation of microglia may lead to the chronic presence of neuroinflammation, which can have detrimental effects on the nervous system. Therefore, ameliorating neuroinflammation and preventing excessive microglial activation may contribute to slowing the progression of AD. The IBA1 protein is highly expressed in microglia and is commonly used as a marker for these cells, being one of the most widely used markers in immunohistochemical analysis of microglia.

DSS is a traditional Chinese medicinal formula with a millennium of usage experience, demonstrating significant therapeutic efficacy in the treatment of inflammation [29–31]. Our results indicate that following the intracerebral injection of Aβ_1−42_ to establish a model in mice, there was a significant increase in the expression of IBA1, a microglial activation marker, suggesting that Aβ_1−42_ induced the activation of microglia. Administration of the DSS, SG, XG, and Donepezil led to the suppression of microglial activation, as evidenced by reduced IBA1 expression. Concurrently, the expression of inflammatory cytokines decreased, indicating that the treatment groups could alleviate neuroinflammation in the AD model.

Studies have shown that intracerebral injection of Aβ_1−42_ can cause neuronal damage and synaptic dysfunction due to abnormal expression of PSD-95 [32]. PSD-95 is an important postsynaptic protein involved in the regulation of synapse formation, function, and plasticity, predominantly located in the dense area of the postsynaptic membrane. HE and Western blot results revealed that DSS, SG, and XG improved the pathological morphological damage of neurons and restored the expression of PSD-95.

The amyloid precursor protein (APP) undergoes sequential degradation by β-proteases and γ-proteases, resulting in the production of Aβ. In this study, all administration groups, except the SG, were able to partially reverse the increase of APP induced by Aβ injection. Notably, the DSS displayed better efficacy in reducing APP. As previously mentioned, deposition of Aβ accelerates Tau phosphorylation, leading to neurofibrillary tangles, while aberrant Tau phosphorylation increases Aβ production by trapping endosomes containing APP [33]. We discovered that p-Tau could be reduced in all administration groups, with the DSS showing better efficacy compared to the SG and XG.

Impaired autophagy can also contribute to the exacerbation of AD [6]. After discovering that every treatment group could restore pathological indicators related to AD to different extents, we aimed to verify the potential association between this neuroprotective effect and autophagy. Therefore, we analyzed Beclin1, P62, and LC3 protein expression. The process of autophagy involves multiple proteins, among which LC3 is essential and considered to be the key marker protein in the cellular autophagy signaling pathway. During autophagy, LC3-I is modified and processed into LC3-II, which attaches to the autophagosome membrane [34]. P62 is an important selective autophagy adaptor protein that serves as a bridge between LC3 and polyubiquitinated proteins. Due to being degraded by proteases in the lysosome along with its substrate, the expression level of the p62 protein is negatively correlated with autophagic activity [35]. Beclin1 is associated with the extension of autophagosomes, which can promote the occurrence of autophagy. Relevant studies have shown that it plays a crucial role in coordinating the cellular protective function of autophagy and opposing the cell death process of apoptosis [36]. Our research findings revealed that after intracerebral injection of Aβ in mice, the expression of LC3 and Beclin1 is decreased, while the expression of P62 is increased. However, after administering DSS, the expression levels of these three proteins are reversed, indicating that DSS can reverse the autophagy inhibition caused by Aβ.

The process of autophagy is related to a variety of cellular regulatory factors, among which the AMPK/mTOR signaling pathway holds a significant role. AMPK and mTOR constitute a pivotal pair of cellular signals that sense energy and nutrition availability and also participate in autophagy [37]. AMPK acts as a positive regulator of autophagy, while mTOR functions as a negative regulator. In our previous studies, paeoniflorin, ferulic acid, and atractylenolide III were found to enhance the AMPK-related autophagy signaling pathway and improve neuroinflammation in BV2 microglia [38, 39]. Meanwhile, additional research has shown that treating AD benefits by activating the AMPK/mTOR autophagy signaling pathway. For example, magnolol enhances AD-related pathophysiology by suppressing cell apoptosis and activating autophagy through the activation of the AMPK/mTOR/ULK1 pathway [40]. Genipin reduces p-Tau levels and Aβ production by triggering the AMPK/mTOR signaling pathway [41]. Furthermore, Dihydrotanshinone I enhanced autophagy via the AMPK/mTOR pathway, reducing p-Tau and enhancing Aβ clearance [42]. Consistent with the previously mentioned findings, our study showed a decrease in p-AMPK expression and an increase in p-mTOR expression within the model group. Conversely, after DSS was administered, p-AMPK expression increased and p-mTOR expression decreased, suggesting that DSS triggered the AMPK/MTOR autophagy pathway.

## Conclusions

In conclusion, DSS demonstrated potential for treating AD by reducing inflammation, thereby restoring neuronal function, and lowering the levels of APP and p-Tau proteins. The AMPK/mTOR autophagy pathway may have been activated to mediate these effects. Additionally, in the research of disassembled prescriptions, the whole prescription of DSS showed better effectiveness for treatment, which reflected the ingenious point of the traditional Chinese medicine formula theory. While this study provided valuable insights, it also had certain limitations that need to be acknowledged and addressed in future research. Firstly, the selection of drug dosages did not include an exploration of dose-response relationships. Secondly, further in-depth reverse validation is required to confirm its role in the neuroprotective effects of DSS, SG, and XG. Additionally, the assessment of neuronal and synaptic recovery was based on a limited set of indicators. Future studies should incorporate a broader range of markers to validate the extent and nature of recovery following treatment. Lastly, this study utilized only the Aβ intracerebral injection model. To enhance the validity of our findings, future research should extend to other models, such as transgenic models of AD, to provide a more comprehensive understanding of the impacts of DSS, SG, and XG.

### Electronic supplementary material

Below is the link to the electronic supplementary material.

Supplementary Material 1

Supplementary Material 2

## Data Availability

The datasets used and/or analyzed during the current study are available from the corresponding author upon reasonable request.

## Abbreviations

AD: Alzheimer’s disease
DSS: Danggui Shaoyao San
SG: Suangan group
XG: Xingan group
Mod: Model
Aβ: Amyloid-β protein
APP: Amyloid precursor protein
p-Tau: Phosphorylated microtubule-associated protein tau
PSD-95: Postsynaptic density protein 95
TCM: Traditional Chinese medicine
ICV: Intracerebroventricularly injected
RT-qPCR: Real-Time Quantitative polymerase chain reaction
ELISA: Enzyme-linked immunosorbent assay
HE: Hematoxylin-eosin staining
IL-1β: Interleukin beta 1,IL-6,interleukin-6
TNF-α: Tumor necrosis factor-alpha
IBA1: Ionized calcium-binding adapter molecule 1
LC3B: Microtubule-associated protein 1 light chain 3 beta
AMPK: Adenosine 5′-monophosphate (AMP)-activated protein kinase
mTOR: Mammalian target of rapamycin
P62: Sequestosome-1
GAPDH: Glyceraldehyde phosphate dehydrogenase
