# Supplementary material for: Danggui Shaoyao San and disassembled prescription: neuroprotective effects via AMPK/mTOR-mediated autophagy in mice
Source: BMC Complement Med Ther. 2024 Aug 10;24:298. doi: 10.1186/s12906-024-04588-x (PMC11317013; doi:10.1186/s12906-024-04588-x)
Supplement: Supplementary file 1 — Supplementary Material 1 [file 12906_2024_4588_MOESM1_ESM.docx]

**Table S1 Basic Information of Active Components in Angelica and Paeonia Powder**

| 中药名 | 编号 | 标识 | 分子名称 | OB (%) | DL |
| --- | --- | --- | --- | --- | --- |
| DG | A1 | MOL000358 | beta-sitosterol | 36.91 | 0.75 |
|  | DG1 | MOL000449 | Stigmasterol | 43.82 | 0.75 |
|  | DG2 | MOL000360 | ferulic acid | 39.55 | 0.05 |
| BS | BS1 | MOL001918 | paeoniflorgenone | 87.59 | 0.36 |
|  | BS2 | MOL001919 | (3S,5R,8R,9R,10S,14S)-3,17-dihydroxy-4,4,8,10,14-pentamethyl-2,3,5,6,7,9-hexahydro-1H-cyclopenta[a]phenanthrene-15,16-dione | 43.55 | 0.53 |
|  | BS3 | MOL001921 | Lactiflorin | 49.12 | 0.79 |
|  | BS4 | MOL001924 | paeoniflorin | 53.87 | 0.78 |
|  | BS5 | MOL001925 | paeoniflorin_qt | 68.17 | 0.39 |
|  | BS6 | MOL000211 | Mairin | 55.37 | 0.77 |
|  | A1 | MOL000358 | beta-sitosterol | 36.91 | 0.75 |
|  | B1 | MOL000359 | sitosterol | 36.91 | 0.75 |
|  | BS7 | MOL000422 | kaempferol | 41.88 | 0.24 |
|  | BS8 | MOL000492 | (+)-catechin | 54.82 | 0.24 |
| CX | CX1 | MOL001494 | Mandenol | 41.99 | 0.19 |
|  | CX2 | MOL002135 | Myricanone | 40.59 | 0.51 |
|  | CX3 | MOL002140 | Perlolyrine | 65.94 | 0.27 |
|  | CX4 | MOL002151 | senkyunone | 47.66 | 0.24 |
|  | CX5 | MOL002157 | wallichilide | 42.31 | 0.7 |
|  | B1 | MOL000359 | sitosterol | 36.91 | 0.75 |
|  | CX6 | MOL000433 | FA | 68.96 | 0.7 |
| BZ | BZ1 | MOL000020 | 12-senecioyl-2E,8E,10E-atractylentriol | 62.39 | 0.22 |
|  | BZ2 | MOL000021 | 14-acetyl-12-senecioyl-2E,8E,10E-atractylentriol | 60.31 | 0.3 |
|  | BZ3 | MOL000022 | 14-acetyl-12-senecioyl-2E,8Z,10E-atractylentriol | 63.37 | 0.29 |
|  | BZ4 | MOL000028 | α-Amyrin | 39.51 | 0.76 |
|  | BZ5 | MOL000033 | (3S,8S,9S,10R,13R,14S,17R)-10,13-dimethyl-17-[(2R,5S)-5-propan-2-yloctan-2-yl]-2,3,4,7,8,9,11,12,14,15,16,17-dodecahydro-1H-cyclopenta[a]phenanthren-3-ol | 36.22 | 0.78 |
|  | BZ6 | MOL000049 | 3β-acetoxyatractylone | 54.06 | 0.21 |
|  | BZ7 | MOL000072 | 8β-ethoxy atractylenolide Ⅲ | 35.95 | 0.21 |
|  | BZ8 | MOL000045 | atractylenolide Ⅲ | 68.11 | 0.17 |
| FL | FL1 | MOL000273 | (2R)-2-[(3S,5R,10S,13R,14R,16R,17R)-3,16-dihydroxy-4,4,10,13,14-pentamethyl-2,3,5,6,12,15,16,17-octahydro-1H-cyclopenta[a]phenanthren-17-yl]-6-methylhept-5-enoic acid | 30.93 | 0.81 |
|  | FL2 | MOL000275 | trametenolic acid | 38.71 | 0.8 |
|  | FL3 | MOL000276 | 7,9(11)-dehydropachymic acid | 35.1 | 0.81 |
|  | FL4 | MOL000279 | Cerevisterol | 37.96 | 0.77 |
|  | FL5 | MOL000280 | (2R)-2-[(3S,5R,10S,13R,14R,16R,17R)-3,16-dihydroxy-4,4,10,13,14-pentamethyl-2,3,5,6,12,15,16,17-octahydro-1H-cyclopenta[a]phenanthren-17-yl]-5-isopropyl-hex-5-enoic acid | 31.07 | 0.81 |
|  | FL6 | MOL000282 | ergosta-7,22E-dien-3beta-ol | 43.5 | 0.71 |
|  | FL7 | MOL000283 | Ergosterol peroxide | 40.36 | 0.81 |
|  | FL8 | MOL000285 | (2R)-2-[(5R,10S,13R,14R,16R,17R)-16-hydroxy-3-keto-4,4,10,13,14-pentamethyl-1,2,5,6,12,15,16,17-octahydrocyclopenta[a]phenanthren-17-yl]-5-isopropyl-hex-5-enoic acid | 38.25 | 0.82 |
|  | FL9 | MOL000287 | 3beta-Hydroxy-24-methylene-8-lanostene-21-oic acid | 38.69 | 0.8 |
|  | FL10 | MOL000289 | pachymic acid | 33.62 | 0.81 |
|  | FL11 | MOL000290 | Poricoic acid A | 30.6 | 0.76 |
|  | FL12 | MOL000291 | Poricoic acid B | 30.52 | 0.74 |
|  | FL13 | MOL000292 | poricoic acid C | 38.15 | 0.74 |
|  | FL14 | MOL000296 | hederagenin | 36.91 | 0.75 |
|  | FL15 | MOL000300 | dehydroeburicoic acid | 44.17 | 0.83 |
| ZX | B1 | MOL000359 | sitosterol | 36.91 | 0.75 |
|  | ZX1 | MOL000830 | Alisol B | 34.47 | 0.81 |
|  | ZX2 | MOL000831 | Alisol B monoacetate | 35.57 | 0.8 |
|  | ZX3 | MOL000832 | alisol,b,23-acetate | 32.51 | 0.81 |
|  | ZX4 | MOL000849 | 16β-methoxyalisol B monoacetate | 32.42 | 0.76 |
|  | ZX5 | MOL000853 | alisol B | 36.76 | 0.81 |
|  | ZX6 | MOL000854 | alisol C | 32.7 | 0.81 |
|  | ZX7 | MOL000856 | alisol C monoacetate | 33.06 | 0.82 |
|  | ZX8 | MOL002464 | 1-Monolinolein | 37.17 | 0.3 |
|  | ZX9 | MOL000862 | [(1S,3R)-1-[(2R)-3,3-dimethyloxiran-2-yl]-3-[(5R,8S,9S,10S,11S,14R)-11-hydroxy-4,4,8,10,14-pentamethyl-3-oxo-1,2,5,6,7,9,11,12,15,16-decahydrocyclopenta[a]phenanthren-17-yl]butyl] acetate | 35.57 | 0.8 |
