# Supplementary figures and images for: Danggui Shaoyao San and disassembled prescription: neuroprotective effects via AMPK/mTOR-mediated autophagy in mice
Source: BMC Complement Med Ther. 2024 Aug 10;24:298. doi: 10.1186/s12906-024-04588-x (PMC11317013; doi:10.1186/s12906-024-04588-x)

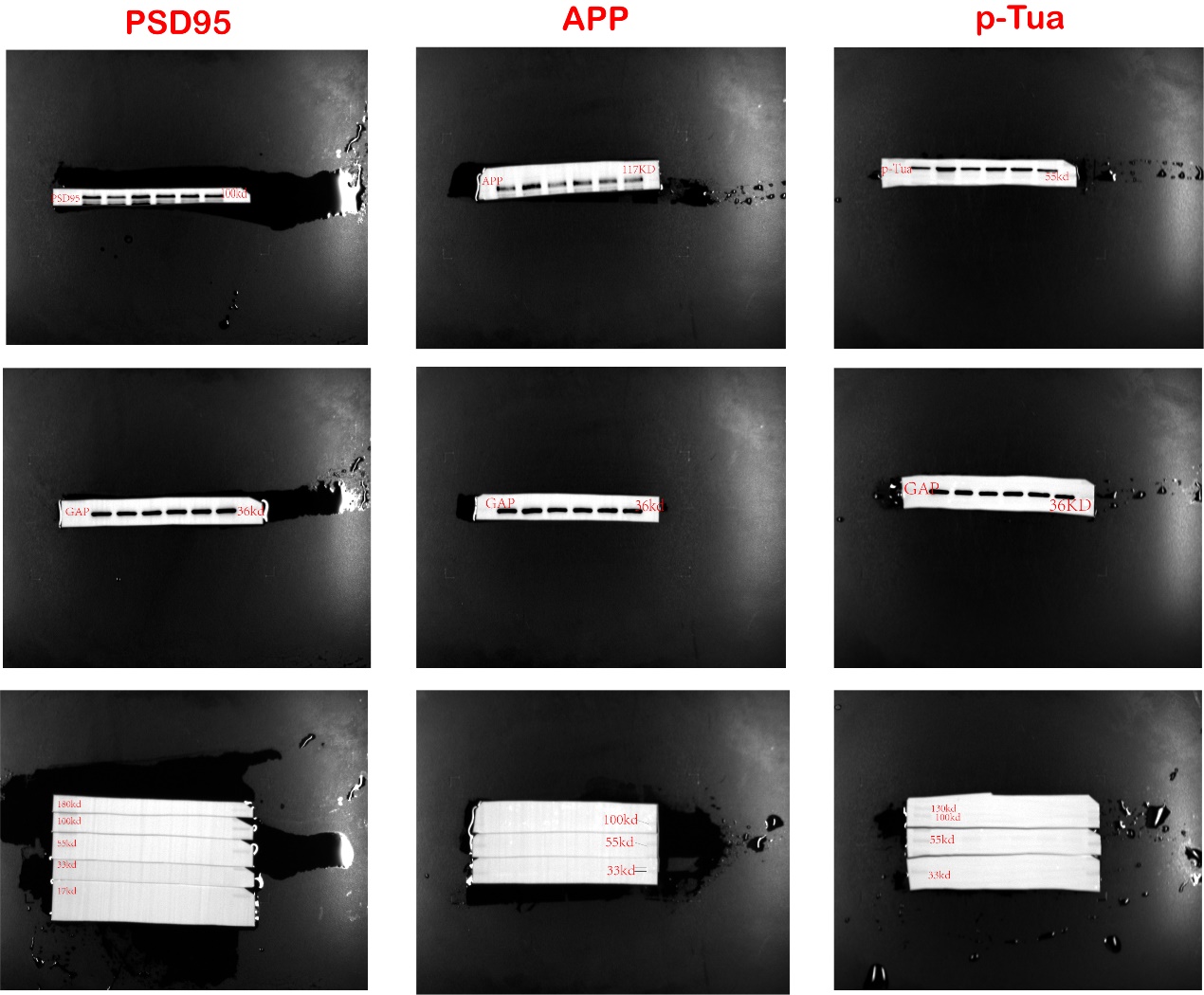


Supplementary Figure 1


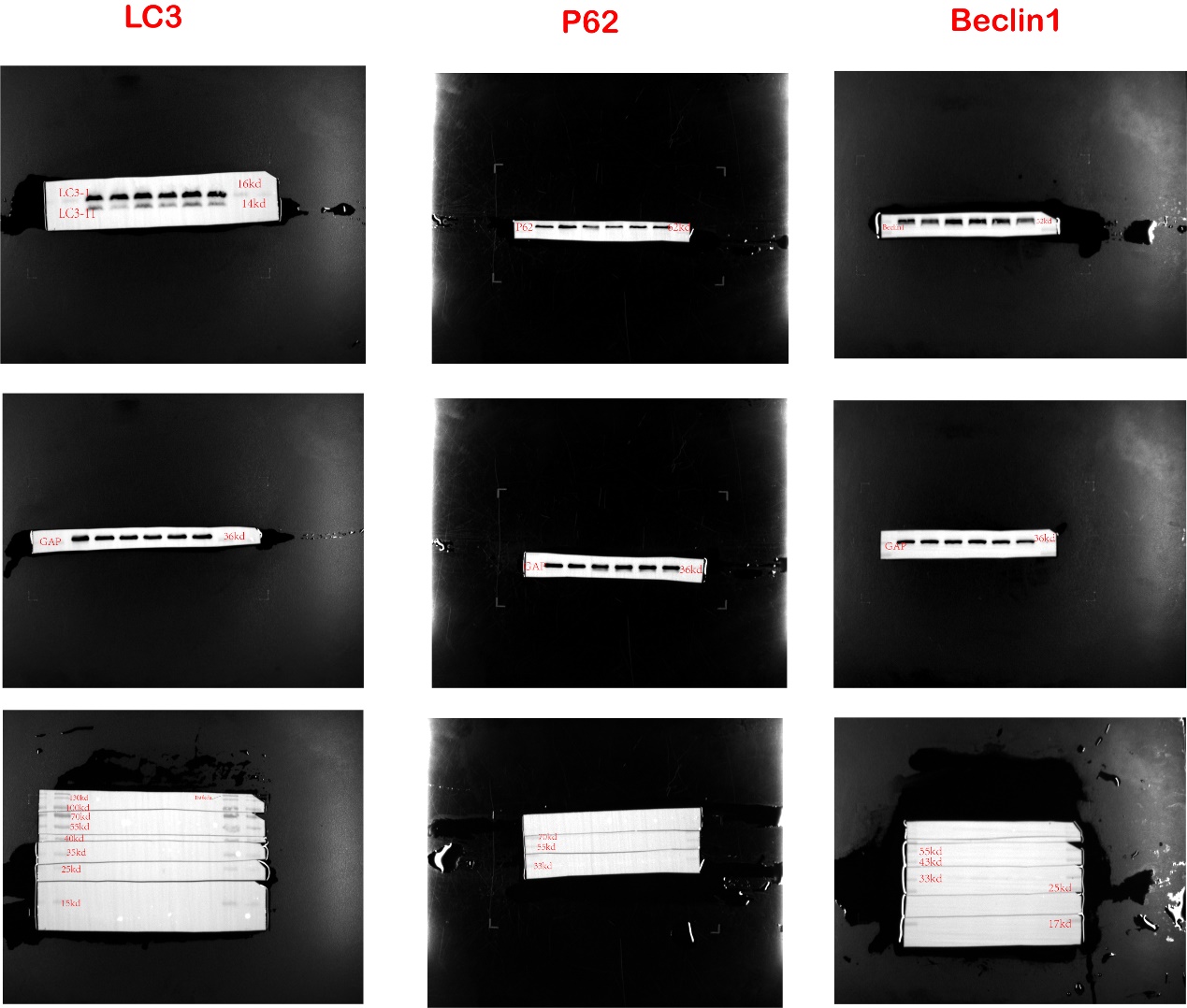


Supplementary Figure 2


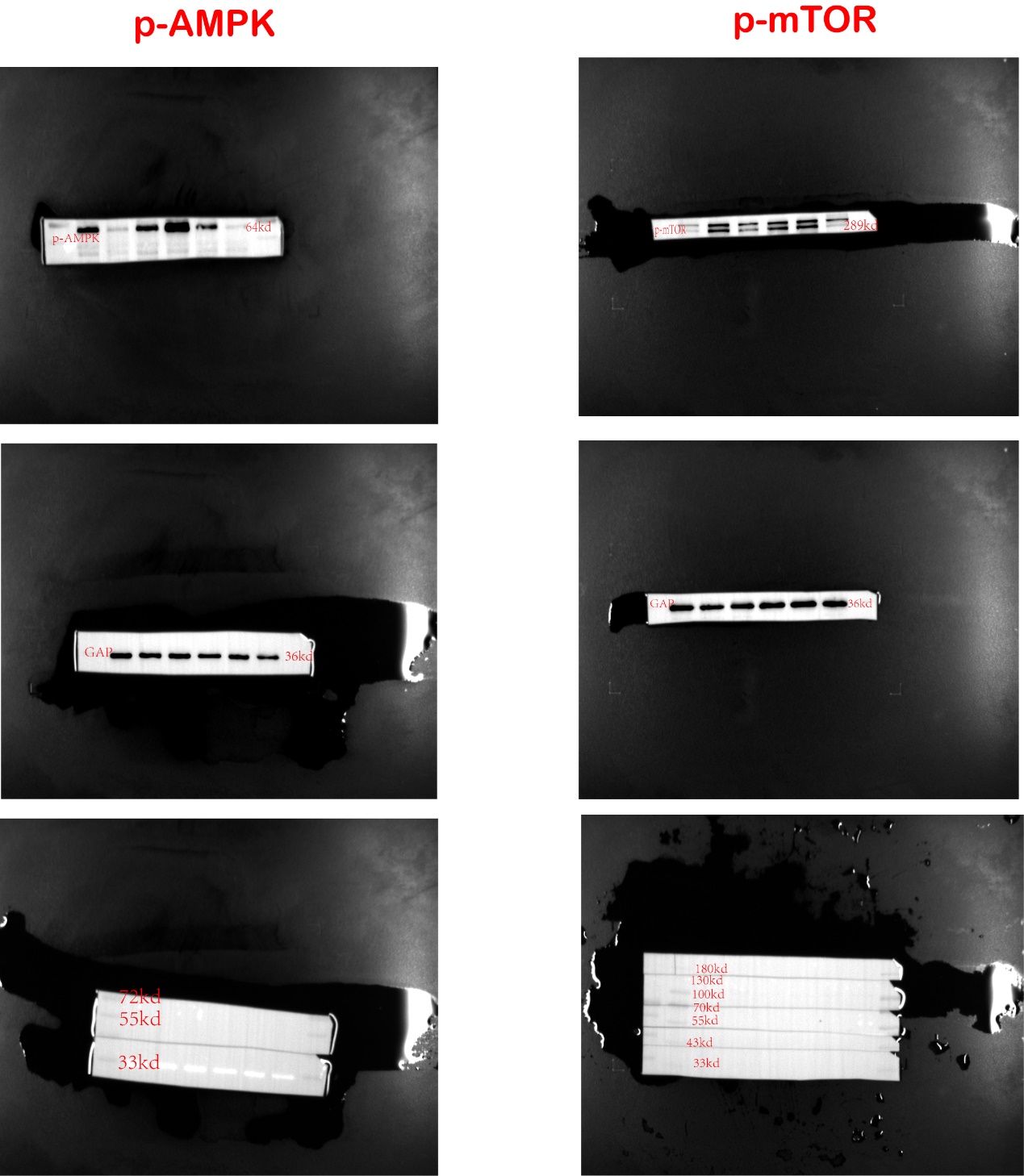


Supplementary Figure 3

Supplement: Supplementary file 2 — Supplementary Material 2 [file 12906_2024_4588_MOESM2_ESM.docx]
